# Long intergenic non-coding RNA 00152 promotes lung adenocarcinoma proliferation via interacting with EZH2 and repressing IL24 expression

**DOI:** 10.1186/s12943-017-0581-3

**Published:** 2017-01-21

**Authors:** Qin-nan Chen, Xin Chen, Zhen-yao Chen, Feng-qi Nie, Chen-chen Wei, Hong-wei Ma, Li Wan, Shuai Yan, Sheng-nan Ren, Zhao-xia Wang

**Affiliations:** 1 Department of Oncology, Second Affiliated Hospital, Nanjing Medical University, Nanjing, 210011 People’s Republic of China; 2 Department of Pathology, First Affiliated Hospital, Nanjing Medical University, Nanjing, People’s Republic of China; 3 Department of Oncology, Huai’an First People’s Hospital, Nanjing Medical University, Nanjing, People’s Republic of China; 4 Department of Oncology, The Affiliated Jiangyin Hospital Of Southeast University Medical College, Jiangyin, China; 5 Department of Oncology, Sir Run Run Hospital, Nanjing Medical University, Nanjing, People’s Republic of China

**Keywords:** Long intergenic non-coding RNA, LINC00152, Lung adenocarcinoma, Proliferation, IL24

## Abstract

**Background:**

Numerous studies have shown that long non-coding RNAs (lncRNAs) behave as a novel class of transcript during multiple cancer processes, such as cell proliferation, apoptosis, migration, and invasion. LINC00152 is located on chromosome 2p11.2, and has a transcript length of 828 nucleotides. The biological role of LINC00152 in LAD(lung adenocarcinoma) remains unknown.

**Methods:**

Quantitative reverse transcription PCR(qRT-PCR) was used to detect LINC00152 expression in 60 human LAD tissues and paired normal tissues. In vitro and in vivo studies showed the biological function of LINC00152 in tumour progression. RNA transcriptome sequencing technology was performed to identify the downstream suppressor IL24(interleukin 24) which was further examined by qRT-PCR, western bolt and rescue experiments. RNA immunoprecipitation (RIP), RNA pulldown, and Chromatin immunoprecipitation (ChIP) assays were carried out to reveal the interaction between LINC00152, EZH2 and IL24.

**Results:**

LINC00152 expression was upregulated in 60 human LAD tissues and paired normal tissues. High levels of LINC00152 expression were correlated with advanced TNM stage, larger tumor size, and lymph node metastasis, as well as shorter survival time. Silencing of LINC00152 suppressed cell growth and induced cell apoptosis. LINC00152 knockdown altered the expression of many downstream genes, including IL24. LINC00152 could interact with EZH2 and inhibit IL24 transcription. Moreover, the ectopic expression of IL24 repressed cell proliferation and partly reversed LINC00152 overexpression-induced promotion of cell growth in LAD.

**Conclusions:**

Our study reveals an oncogenic role for LINC00152 in LAD tumorigenesis, suggesting that it could be used as a therapeutic target in LAD treatment.

**Electronic supplementary material:**

The online version of this article (doi:10.1186/s12943-017-0581-3) contains supplementary material, which is available to authorized users.

## Background

Non-small cell lung cancer (NSCLC) accounts for 80% of all lung cancer cases [1]. The incidence of NSCLC has increased annually, and it is becoming one of the leading causes of death from cancer worldwide. Lung adenocarcinoma (LAD) is the most common histological type of NSCLC, and because of its tendency to form haematogenous metastasis, patients often die of relapse and metastasis [2]. Although advanced detection measures and treatments including surgery, chemotherapy, radiotherapy, and targeted therapy are widely used, the likelihood of a complete cure is slim [3, 4]. Therefore, a thorough understanding of the molecular mechanisms involved in the development and progression of LAD could provide more effective diagnostic markers and targets for LAD patient therapy [5].

The completion of the human genome and GENCODE projects revealed that less than 3% of the human genome codes for proteins, while at least 75% is transcribed into non-coding RNAs, including microRNAs (miRNAs) (<200 nt) and long non-coding RNAs (lncRNAs) (>200 nt). Many studies have shown that miRNAs can act either as tumor suppressors or oncogenes in various cancers through regulating target gene expression by binding 3′ untranslated regions and repressing transcription [6, 7]. Recently, lncRNAs have drawn attention as potential biological regulators involved in a wide range of cellular activities such as cell proliferation, apoptosis, migration, and invasion [8]. The underlying mechanisms of lncRNAs are varied, and include acting as competitive endogenous RNAs similar to miRNA sponges, chromatin remodelling, and histone protein modification [9]. Moreover, the dysregulation of lncRNAs has been linked with human diseases including cancers [10]. For example, the SP-1-induced overexpression of tissue differentiation-inducing non-protein coding RNA was previously shown to promote cell proliferation by influencing KLF2 mRNA stability in gastric cancer [11]. Nie et al. also reported that UCA1 competitively sponged miR193a-3p and induced expression of the miR-193a-3p target gene ERBB4 in NSCLC [12]. Additionally, in an earlier study, we found that the long non-coding RNA PVT1 suppressed cell growth and induced apoptosis by binding to the enhancer of zeste homolog 2 (EZH2) protein, a histone methyltransferase of the PRC2 complex, in LAD [13].

Long intergenic non-coding RNA 00152 (LINC00152) is located on chromosome 2p11.2, and has a transcript length of 828 nucleotides. It was first reported to be highly expressed in gastric tissues and cells [14]. Further work showed that LINC00152 was involved in cell proliferation, apoptosis, migration, and invasion in gastric cancer and clear cell renal cell cancer, suggesting that it could be viewed as a potential target in both cancers [15–17]. LINC00152 was also observed to act as a novel biological predictor in hepatocellular carcinoma (HCC), being positively correlated with advanced TNM stage and poor overall survival [18]. However, the expression pattern, functional role, underlying mechanism, and clinical significance of LINC00152 in LAD remain unclear.

In this study, we evaluated the expression level of LINC00152 in LAD tissues and cells by analyzing gene profiling data from the TCGA database and validating these in a cohort of 60 paired tissues. We found that LINC00152 was significantly up-regulated in LAD tissues compared with normal lung tissues, and that increased LINC00152 levels were associated with poor prognosis and short survival time of LAD patients. LINC00152 was also shown to bind to PRC2 protein, thereby inhibiting the expression of interleukin (IL)24 and promoting LAD cell proliferation.

## Methods

### Tissue samples and clinical data collection

A total of 60 paired LAD and adjacent non-tumor lung tissues were obtained from patients who underwent surgery at The First and Second Affiliated Hospital of Nanjing Medical University from 2012 to 2013. All patients were diagnosed with LAD (stages I, II, and III) according to histopathological evaluation. Clinicopathological characteristics of LAD patients are shown in Table 1. No chemotherapy or radiotherapy was conducted in these patients prior to surgery. All tissues were stored at −80°C. All human tissue samples were obtained with written informed consent from all subjects, and this project was approved by the Research Ethics Committee of The Second Affiliated Hospital of Nanjing Medical University. All methods were carried out in accordance with the guidelines approved by the Research Ethics Committee of The Second Affiliated Hospital of Nanjing Medical University. And the study protocol was approved by the Research Ethics Committee of The Second Affiliated Hospital of Nanjing Medical University.

**Table 1:** Relationship between LINC00152 expression and clinicopathological characteristics of LAD patients

Characteristics	N (%)	linc00152	linc00512	*P*
		High	Low	Chi-square
				d-test
				*P*-value
Age (years)				0.436
> 65	27 (45%)	12	15	
≤ 65	33 (55%)	18	15	
Gender				0.598
Male	24 (40%)	13	11	
Female	36 (60%)	17	19	
TNM stage				0.035^*^
I + II	24 (40%)	8	16	
IIIa	36 (60%)	22	14	
Tumor size				0.020^*^
≤ 5	16 (26.7%)	4	12	
> 5	44 (73.3%)	26	18	
Lymph node metastasis				0.001^*^
Negative	22 (36.7%)	5	17	
Positive	38 (63.3%)	25	13	
Smoking history				0.301
Smokers	28 (46.7%)	16	12	
Never smoker	32 (53.3%)	14	18	

*P < 0.05

### Cell culture

We obtained five LAD cell lines (A549, SPCA1, PC-9, H1299, and H1975) and the normal human bronchial epithelial cell line 16HBE from the Institute of Biochemistry and Cell Biology of the Chinese Academy of Sciences (Shanghai, China). A549, H1975, and H1299 cells were cultured in RPMI-1640 medium (GIBCO-BRL), and 16HBE, SPC-A1, and PC9 cells were grown in DMEM medium (GIBCO-BRL). Both media were supplemented with 10% fetal bovine serum (FBS; Gibco) and antibiotics (100 U/ml penicillin and 100 mg/ml streptomycin) (Invitrogen, Carlsbad, CA) were maintained in a humidified air atmosphere at 37°C with 5% CO_2_.

### RNA isolation and qRT-PCR

Total RNA was extracted from tissues or cultured cells using TRIzol reagent (Invitrogen). Total RNA (1 μg) was reverse transcribed to cDNA in a final volume of 20 μl using random primers under standard conditions with the PrimeScript RT Reagent Kit (Takara, Dalian, China). We performed real-time PCR analyses using SYBR Premix Ex Taq (Takara) according to the manufacturer’s instructions. Results were normalised to the expression of glyceraldehyde 3-phosphate dehydrogenase (GAPDH), and data were collected based on the comparative cycle threshold (CT) (2^−ΔΔCT^) method. Specific primer sequences are listed in Additional file 1: Table S2.

### RNA interference

A549 and SPCA1 cell lines were seeded in six-well plates, then 24 h later they were transfected with specific siRNAs and plasmid vectors using Lipofectamine 2000. We purchased three LINC00152 siRNAs (si-LINC00152 1#, 2#, and 3#), EZH2 siRNA, and scrambled negative control siRNA (si-NC) from Invitrogen. LINC00152 and EZH2 siRNA sequences are listed in Additional file 1: Table S2. Cells were harvested for qRT-PCR or western blot analysis 48 h after transfection.

### Plasmid generation

Full-length LINC00152 cDNA was synthesised by Realgene (Nanjing, China) and ligated into the pcDNA3.1(+) vector (Invitrogen). The IL24 sequence was also synthesised and subcloned into the pCDNA3.1(+) vector (GENECHEM, Shanghai, China). Plasmid vectors (pcDNA3.1-LINC00152, pcDNA3.1-IL24, and empty vector) were transfected into LAD cells cultured in six-well plates using the X-tremeGENE HP DNA transfection reagent (Roche, Basel, Switzerland). Cells were harvested for qRT-PCR or western blot analysis 48 h after transfection.

### Cell proliferation assays

Cell viability was measured using the Cell Proliferation Reagent Kit I (MTT; Roche Applied Science). A549 and SPCA1 cells transfected with si-LINC00152, and PC-9 cells transfected with pCDNA-LINC00152 were seeded in 96-well plates. Cell viability was monitored every 24 h following the manufacturer’s instructions. For the colony formation assay, a total of 1 × 10^3^ transfected cells were placed in each well of 6-well plates and maintained in media containing 10% FBS for 2 weeks, during which the medium was replaced every 3 days. After 14 days, the colonies were treated with methanol and stained with 0.1% crystal violet (Sigma-Aldrich). Visible colonies were counted. Wells were assessed in triplicate for each treatment group.

### Flow cytometric analysis

A549 and SPCA1 cells transfected with si-LINC00152 were harvested 48 h after transfection by trypsinisation. After double staining with FITC-Annexin V and propidium iodide (PI) using the FITC Annexin V Apoptosis Detection Kit (BD Biosciences) according to the manufacturer’s recommendations, the cells were analysed by flow cytometry (FACScan®; BD Biosciences) equipped with CellQuest software (BD Biosciences). Cells were classified as viable, dead, early apoptotic, and apoptotic, then the relative number of early apoptotic cells was compared with that in cells transfected with control transfectant. Cells for cell cycle analysis were stained with PI using the CycleTEST™ Plus DNA Reagent Kit (BD Biosciences) following the protocol, and analysed by FACScan. The percentage of cells in G0/G1, S, and G2/M phase were counted and compared.

### Western blot assay and antibodies

Cell protein lysates were separated by 10% sodium dodecyl sulphate (SDS) polyacrylamide gel electrophoresis, transferred to 0.22 μm NC membranes (Sigma) and incubated with specific antibodies. The ECL chromogenic substrate was quantified by densitometry (Quantity One software; Bio-Rad). An anti-GAPDH antibody was used as a control, anti-EZH2, and anti-Bcl-xl antibodies (1:1000) were purchased from Cell Signaling Technology, Inc., and anti-IL24 antibodies (1:1000) were purchased from Abcam.

### Ethynyldeoxyuridine (EdU) analysis

The EdU labelling/detection kit (Ribobio, Guangzhou, China) was used to assess cell proliferation. Cells were grown in 96-well plates at 5 × 10^3^ cells/well. Forty-eight hours after transfection, 50 μM EdU labelling media was added to cells and they were incubated for 2 h at 37°C under 5% CO_2_. After treatment with 4% paraformaldehyde and 0.5% Triton X-100, cells were stained with anti-EdU working solution. DAPI was used to label cell nuclei. The percentage of EdU-positive cells was calculated under fluorescent microscopy. Five fields of view were randomly assessed for each treatment group.

### Tumor formation assay in a nude mouse model

Male athymic BALB/c mice (5-weeks-old) were maintained under specific pathogen-free conditions and manipulated according to protocols approved by the Shanghai Medical Experimental Animal Care Commission. A549 cells were stably transfected with sh-LINC00152 and empty vector and harvested from 6-well cell culture plates, washed with phosphate-buffered saline, and re-suspended at a concentration of 1 × 10^8^ cells/ml. A total of 100 μL of suspended cells was subcutaneously injected into a single side of the posterior flank of each mouse. tumor growth was examined every 3 days, and tumor volumes were calculated using the equation V = 0.5 × D × d^2^ where V represents volume; D represents longitudinal diameter; and d represents latitudinal diameter. At 18 days post-injection, mice were euthanised, and the subcutaneous growth of each tumor was examined. This study was carried out in strict accordance with the recommendations in the Guide for the Care and Use of Laboratory Animals of the National Institutes of Health. The protocol was approved by the Committee on the Ethics of Animal Experiments of Nanjing Medical University.

### Subcellular fractionation

The separation of nuclear and cytosolic fractions was performed using the PARIS Kit (Life Technologies, Carlsbad, CA) following the manufacturer’s instructions.

### RNA immunoprecipitation (RIP) assay

RIP was performed using the EZ-Magna RIP kit (Millipore, Billerica, MA) following the manufacturer’s protocol. A549 and SPCA1 cells at 80–90% confluency were scraped off the tissue culture plate, then lysed in complete RIP lysis buffer. A total of 100 μl of whole cell extract was incubated with RIP buffer containing magnetic beads conjugated with antibodies against EZH2, SUZ12, or LSD1 or control IgG (Millipore) for 6 h at 4°C. The beads were washed with wash buffer, then the complexes were incubated with 0.1% SDS/0.5 mg/ml Proteinase K (30 min at 55°C) to remove proteins. The RNA concentration was measured using a NanoDrop spectrophotometer (Thermo Scientific) and its quality was assessed using a bioanalyser (Agilent, Santa Clara, CA). Finally, immunoprecipitated RNA was purified and analysed by qRT-PCR.

### Chromatin immunoprecipitation

A549 and SPCA1 cells were treated with formaldehyde and incubated for 10 min to generate DNA–protein cross-links. Cell lysates were then sonicated to generate chromatin fragments of 200–300 bp and immunoprecipitated with antibodies specific for EZH2, LSD1, H3K27me3, or H3K4me2 (CST) or IgG as a control. Precipitated chromatin DNA was recovered and analysed by qRT-PCR.

### RNA pulldown assays

The pCDNA3.1-LINC00152 vector was cleaved by restriction enzyme *Nru* I and treated with RNase-free DNase I (New England Biolabs). LINC00152 was transcribed from this vector by mMESSAGE mMACHINE T7® Kit (Ambion, USA) and purified using the RNeasy Mini Kit (Qiagen, Valencia, CA) *in vitro*. The 3′ end of LINC00152 was biotin-labelled according to the instructions of the Pierce RNA 3′ End Desthiobiotinylation Kit (Thermo Scientific, USA). One mg of protein from SPCA1 cell extracts was then mixed with 50 pmol of biotinylated RNA, and incubated with 50 μL of magnetic beads for 1 h at 4°C (Thermo Scientific). The RNA–protein complex was isolated from magnetic beads using Biotin Elution Buffer and boiled in SDS buffer for 5 min. The retrieved protein was detected using standard western blotting techniques.

### Immunohistochemical (IHC) analysis

Primary tumors were immunostained for Ki-67 and IL24 as previously described.

### Statistical analysis

The Student’s *t*-test (two-tailed), one-way analysis of variance, and the Mann–Whitney U test were conducted to analyse in vitro and in vivo data by SPSS 17.0 software. *p* values less than 0.05 were considered significant.

## Results

### LINC00152 is overexpressed in LAD tissues

**Fig. 1:**
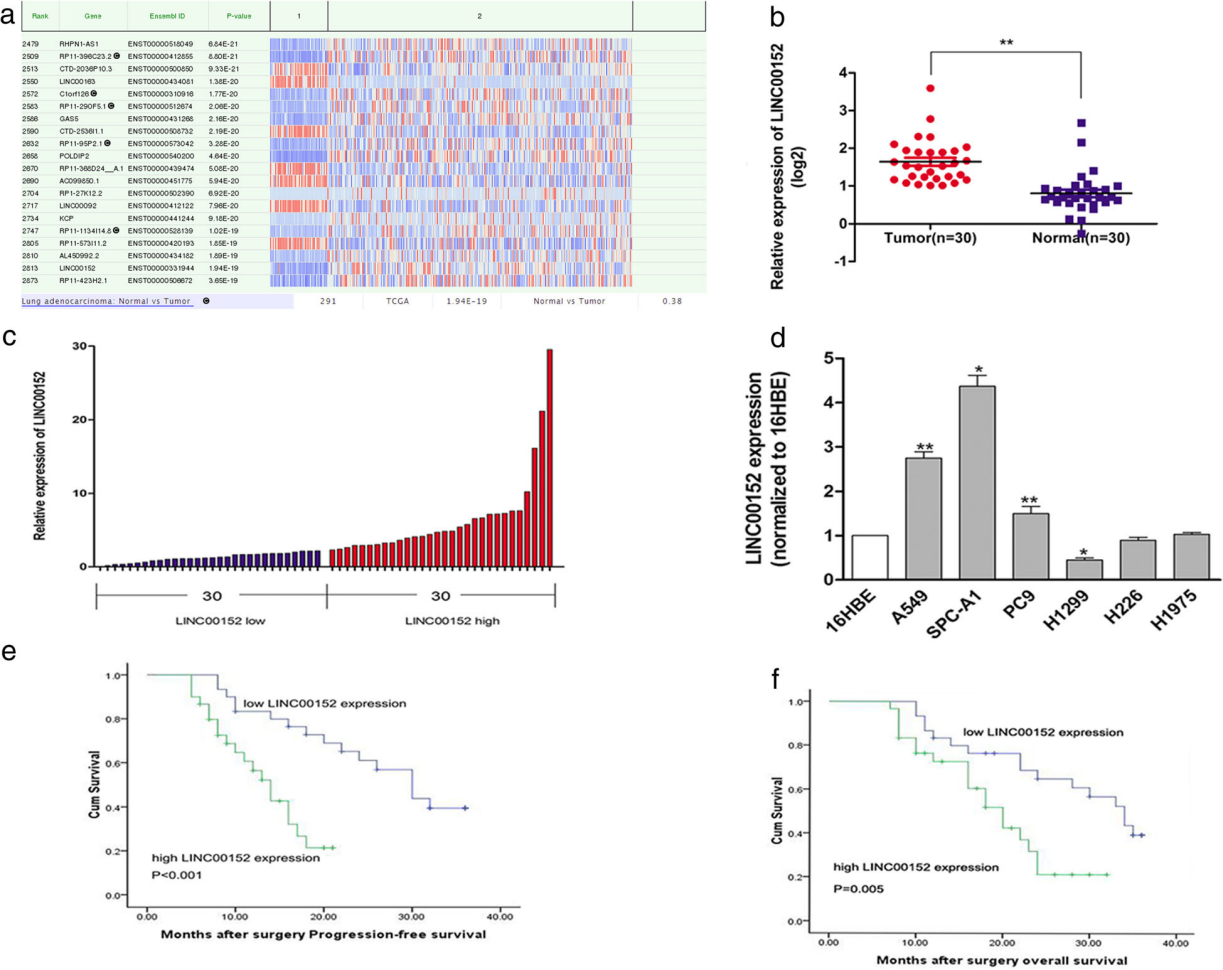
Relative LINC00152 expression in LAD tissues and its clinical significance. **a** Bioinformatics tools “lncRNAtor” (http://lncrnator.ewha.ac.kr/expression.htm) detection of LINC00152 expression in human LAD(*n* = 46) compared with normal tissues(*n* = 245). **b** Relative expression of LINC00152 in LAD tissues compared with non-cancerous tissues analyzed by TCGA database. **c** LINC00152 expression in lung adenocarcinoma (*n* = 60) compared with normal tissues (*n* = 60) was examined by qRT-PCR and normalized to GAPDH expression. **d** LINC00152 expression was analyzed by qRT-PCR in 6 NSCLC cell lines(5 lung adenocarcinoma cell lines and 1 squamous carcinoma cell line), compared with the normal bronchial epithelial cell line(HBE). **e, f** Kaplan-Meier progression-free survival time and overall survival curves according to LINC00152 expression levels. *P < 0.05, **P < 0.01

We first analysed lncRNA expression in LAD tissues (*n* = 291) using the bioinformatics tool “lncRNAtor” (http://lncrnator.ewha.ac.kr/expression.htm), and found that LINC00152 was highly expressed in LAD tumor tissues at ~2.63-fold higher levels than it in normal tissues (Fig. 1a). We also evaluated LINC00152 expression in 30 LAD samples and adjacent normal samples from the Cancer Genome Atlas database. As shown in Fig. 1b (*P* < 0.01), LINC00152 was significantly upregulated in LAD tissues. Quantitative reverse transcription(qRT-PCR) showed that LINC00152 expression was increased in 60 LAD tumor samples compared with paired adjacent normal tissues (Fig. 1c). We also assessed its expression level in 16HBE cells and six human LAD cell lines using qRT-PCR. A549 and SPCA1 cells expressed the highest levels of LINC00152, while H1299 expressed lower LINC00152 levels (Fig. 1d). These results suggested that LINC00152 played an essential biological role in LAD tumorigenesis and progression.

### LINC00152 expression associates with poor prognosis of LAD patients

To assess the significance of LINC00152 overexpression in LAD, we evaluated the correlation between LINC00152 expression and patient clinicopathological characteristics. Sixty LAD patients were classified into two groups according to the median ratio (2.18) of relative LINC00152 expression in tumor tissues: high LINC00152 group (*n* = 30, LINC00152 expression ratio ≤ median ratio) and low LINC00152 group (*n* = 30, LINC00152 expression ratio ≥ median ratio). As shown in Table 1, higher levels of LINC00152 were significantly associated with advanced TNM stage (*p* = 0.035), larger tumor size (*p* = 0.020), and lymph node metastasis (*p* = 0.001). However, there was no obvious relationship between LINC00152 expression and other clinical parameters such as age (*p* = 0.436), sex (*p* = 0.598), or smoking history (*p* = 0.301) (Table 1).

Kaplan–Meier survival analysis was used to examine the association between LINC00152 and LAD patient prognosis. Notably, patients with higher LINC00152 expression levels had significantly shorter progression-free survival time and overall survival time than those expressing lower levels of LINC00152 (*p* = 0.012) (Fig. 1e, f).

### LINC00152 silencing inhibits LAD cell proliferation

To investigate the potential role of LINC00152 in LAD cells, we synthesised three small interfering (si)RNAs to silence LINC00152 expression, and 48 h post-transfection observed knockdown of LINC00152 by 88% in A549 cells and 92% in SPCA1 cells compared with control cells. We also upregulated LINC00152 expression in H1299 cells by 113-fold by transfecting them with the pcDNA3.1-LINC00152 vector (Additional file 2: Figure S1a, b).

**Fig. 2:**
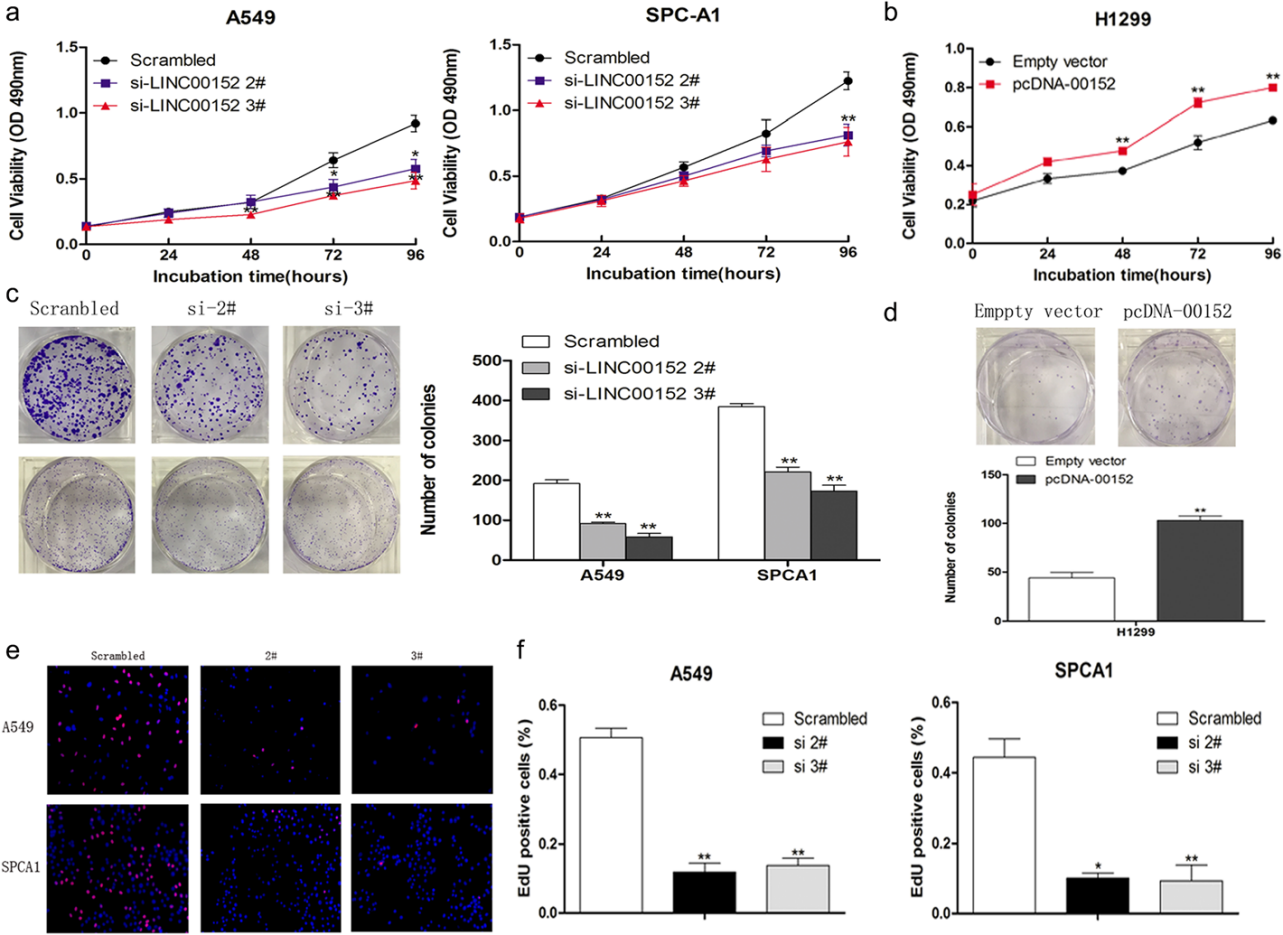
The effects of LINC00152 on LAD cell proliferation in vitro. **a, b** MTT assays were performed to detect the viability of si-LINC00152 transfected A549 and SPCA1 cells or pcDNA-LINC00152 transfected H1299 cells. **c, d** Colony formation assays and EdU immunostaining assays were conducted to determine the proliferation of transfected LAD cells. **e, f** Colonies and EdU positive cells were counted and captured. Values are shown as the mean ± s.d in three independent experiments. *P < 0.05, **P < 0.01

Because lncRNAs are involved in many biological processes, we next examined the contribution of LINC00152 to LAD development. MTT assays showed that A549 and SPCA1 cell viability was significantly decreased after knockdown of LINC00152 expression (Fig. 2a). By contrast, H1299 cells exhibiting increased LINC00152 expression levels showed a higher cell viability rate than controls (Fig. 2b). Additionally, the colony formation ability of A549 and SPCA1 cells was greatly attenuated following LINC00152 knockdown (Fig. 2c), while clonogenic survival was markedly increased in LINC00152-overexpressing H1299 cells (Fig. 2d). Similar results were observed by 5-ethynyl-2-deoxyuridine EdU(red)/DAPI (blue) immunostaining assays (Fig. 2e, f). These findings indicated that LINC00152 behaved as an oncogene promoting LAD cell proliferation.

### Knockdown of LINC00152 induces G1 arrest and cell apoptosis

**Fig. 3:**
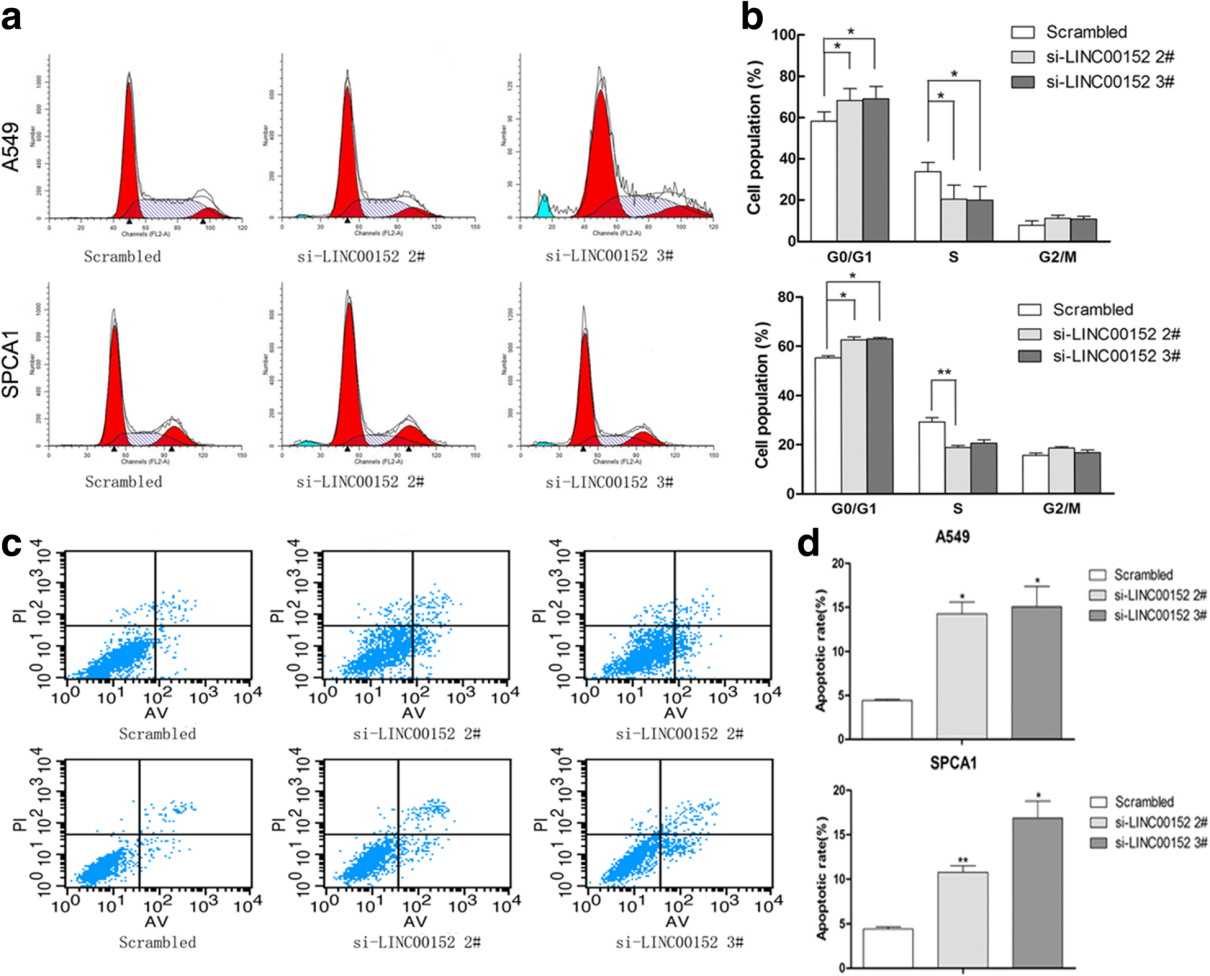
LINC00152 knockdown promoted G1 arrest and inducedd apoptosis in LAD cells. A549 and SPCA1 cells were transfected with scrambled or si-LINC00152 2#, 3#. **a, b** Cell cycle was analyzed in A549 and SPCA1 cells. **c, d** Flow cytometry was performed to determine the apoptptic rates of cells. Values are shown as the mean ± s.d in three independent experiments. *P < 0.05, **P < 0.01

To further explore whether the cell cycle progression alteration contributes to the effect of LINC00152 knockdown on cell viability, flow cytometric analysis was used to examine cell cycle progression. A549 and SPCA1 cells transfected with si-LINC00152 2# or 3# showed an elevated cell cycle arrest at the G1/G0 phase (Fig. 3a, b). Flow cytometric analysis also revealed that LINC00152 knockdown by LINC00152 siRNA increased apoptosis in LAD cells (Fig. 3c, d). Moreover, Bcl-xl protein levels were significantly decreased in cells transfected with siRNA (Additional file 2: Figure S1c). These results confirmed that the LINC00152 downregulation-mediated inhibition of LAD cell proliferation could be attributed to apoptosis and cell cycle arrest at the G1/S checkpoint.

### LINC00152 promotes the tumorigenesis of LAD cells in vivo

**Fig. 4:**
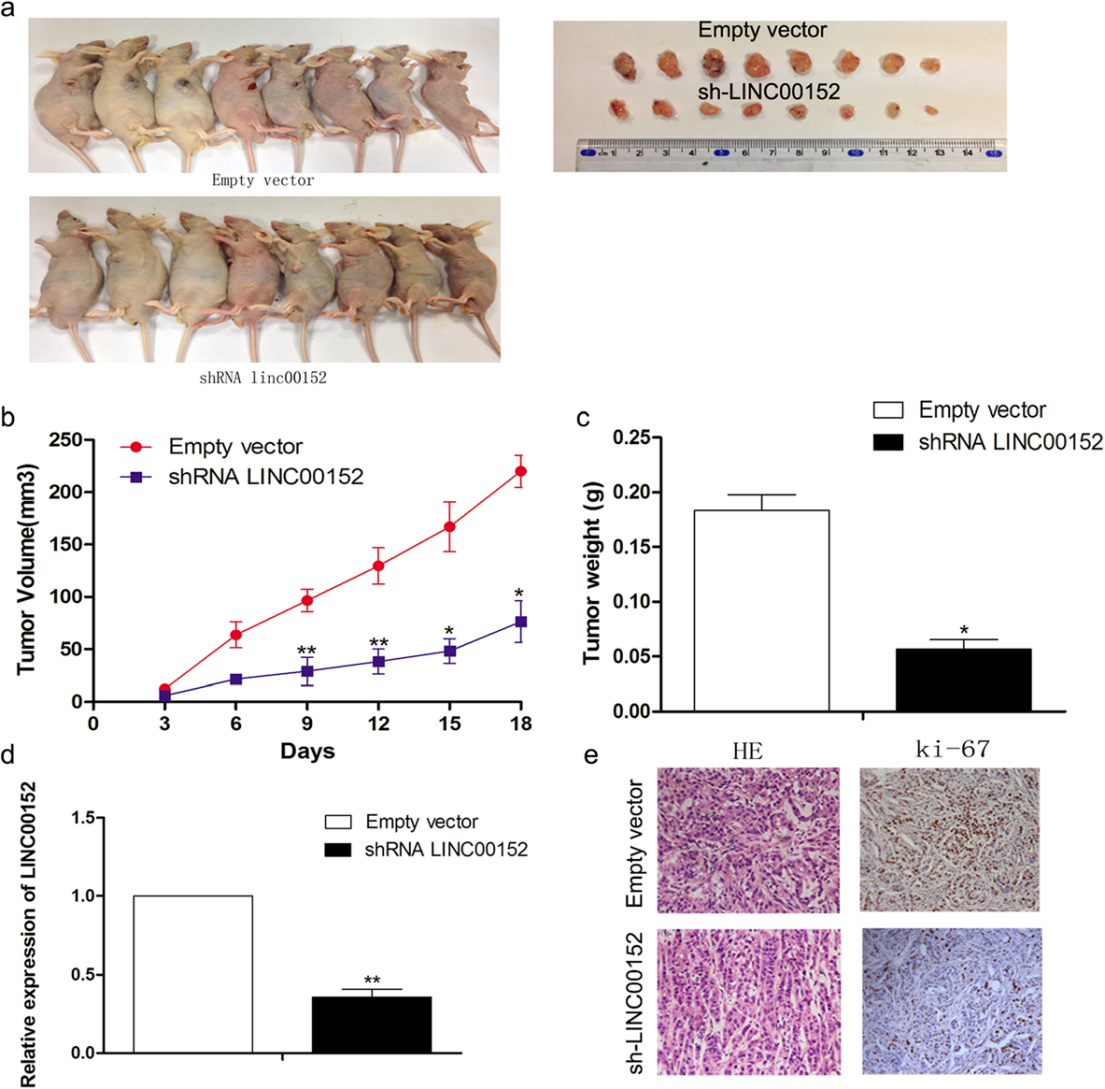
Effects on tumor proliferation after LINC00152 knockdown in vivo. **a** A549 cells transfected with empty vector or sh-LINC00152 were injected into the nude mice(*n* = 8). Tumors before and after carrying from the nude mice. **b** Tumor volume was calculated every 3 days. **c** Tumor weight was measured after tumor removal. **d** qRT-PCR was used to examine the average expression of LINC00152 in tumor tissues formed from A549/empty vector and A549/sh-LINC00152. **e** The tumor sections were under H&E staing and IHC staining using antibodies against Ki-67. *P < 0.05, **P < 0.01

We next constructed a xenograft mouse model to verify the oncogenic role of LINC00152 in LAD tumorigenesis. A549 cells stably transfected with sh-LINC00152 or empty vector (and H1299 cells stably transfected with pcDNA-LINC00152 or empty vector) were injected into nude mice. After 18 days, we found that LINC00152 silencing substantially decreased tumor growth, and that the tumors which formed in the sh-LINC00152 group of mice were smaller than those in the control group (Fig. 4a, b). Additionally, the tumor weight of the sh-LINC00152 group was less than that of the control (Fig. 4c). In contrast, LINC00152 overexpression could promote tumor growth (Additional file 3: Figure S2a, b). qRT-PCR analysis showed that tumor tissues from the sh-LINC00152 group exhibited lower LINC00152 expression than those of the empty vector group (Fig. 4d). Moreover, immunohistochemical analysis revealed decreased Ki-67 staining, reflecting a lower proliferation index, in tumor tissues formed from A549 cells with stable LINC00152 knockdown (Fig. 4e). Collectively, these results indicated that LINC00152 was involved in oncogenic activation in LAD in vivo.

### LINC00152 directly interacted with EZH2/LSD1 in LAD cells

**Fig. 5:**
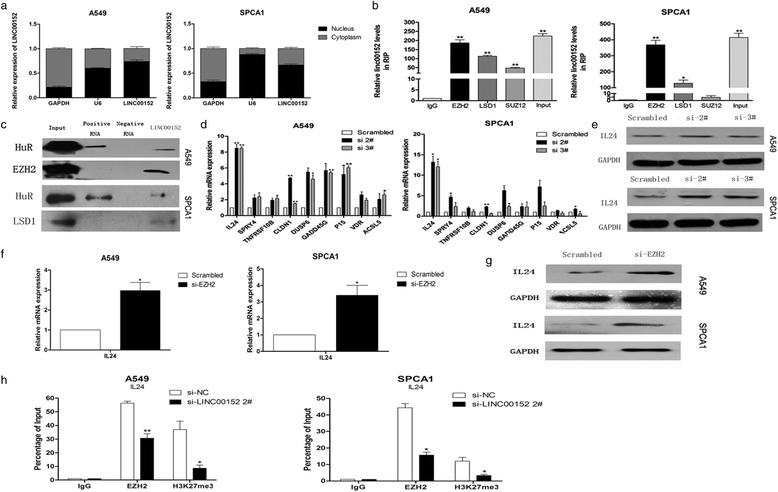
LINC00152 interacted with EZH2, and regulated IL24 expression. **a** qRT-PCR was performed to detect the relative LINC00152 levels in A549 and SPCA1 cell cytoplasm and nucleus. GAPDH was used as cytoplasm control and U6 was used as nucleus control. **b** RIP experiments were performed in A549,SPCA1 cells and the corprecipitated RNA was subjected to qRT-PCR for LINC00152. Expression levels of LINC00152 RNA are as fold enrichment in EZH2,LSD1,SUZ12 relative to IgG immunoprecipitates. **c** RNA pulldown and western blotting assays revealed biotinylated LINC00152 could bind to EZH2 and LSD1. **d** qRT-PCR was used to analyze the mRNA expression levels of tumor suppressor genes in scrambled and si-LINC00152 2#/3# treated LAD cells. **e** Western blotting analysis of IL24 protein level in LINC00152knockdown A549 and SPCA1 cells, GAPDH protein was used as an internal control. **f** IL24 mRNA level in A549 and SPCA1 cells transfected with si-EZH2. **g** Western blotting were performed to detect the IL24 protein level in A549 and SPCA1 cells. **h** ChIP-qRT-PCR of EZH2 occupancy and H3K27me3 binding in the IL24 promoter in A549 and SPCA1 cells treated with si-LINC00152(48h) or scrambled siRNA, IgG was used as a negative control. Values are shown as the mean ± s.d in three independent experiments. *P < 0.05, **P < 0.01

As shown in Fig. 5a, LINC00152 expression in nucleus was higher than it in cytosol in A549 and SPCA1 cell lines, indicating that it may function as a regulator of transcription levels. Previous studies showed that lncRNAs influence the expression of downstream targets via recruiting PRC2 or LSD1 proteins [19, 20]. To determine whether LINC00152 regulates target genes using a similar mechanism, we carried out RNA immunoprecipitation (RIP) assays which revealed that LINC00152 bound directly to EZH2 and LSD1 in both A549 and SPCA1 cells (Fig. 5b). RNA pulldown assays also confirmed the interaction between LINC00152 and EZH2 or LSD1 (Fig. 5c).

### Interleukin (IL)24 is the key underlying target of LINC00152

We used RNA transcriptome sequencing to identify genes that were differentially expressed between LINC00152-depleted SPCA1 cells and control cells. Of 1742 differentially expressed transcripts screened out by database analysis (fold-change >2, *p* < 0.05), 763 were upregulated and 979 were downregulated (Table 2 and Additional file 4: Table S1). We selected nine representative genes verified as tumor suppressors in A549 and SPCA1 cell lines. Of these, IL24 showed the highest fold-change of upregulation in LINC00152-depleted SPCA1 and A549 cells (Fig. 5d). Additionally, western blot analysis showed that IL24 protein levels were increased in LAD cells transfected with LINC00152 (Fig. 5e). qRT-PCR and western blot analysis showed that EZH2 or LSD1 inhibition upregulated the mRNA and protein expression of IL24 (Fig. 5f, g + Additional file 2: Figure S1d, e + Additional file 5: Figure S3a–c).

**Table 2:** Differentially expressed mRNAs in SPCA1 cells transfected with si-linc00152 compared with SPCA1 cells as determined by microarray

mRNAs	Regulation	Ratio	mRNAs	Regulation	Ratio
Il24	Up	10.63981	GPC6	Down	27.78241
GADD45G	Up	8.40941	FGF7	Down	20.73484
HTR6	Up	8.13291	ASTN1	Down	15.33507
NWD2	Up	8.03484	LAMP3	Down	9.11494
GPX7	Up	7.99557	EYA1	Down	8.44694
SHC4	Up	5.52016	RIC3	Down	7.95735
PAGE2	Up	5.12265	GPR85	Down	7.87315
NT5E	Up	4.92725	RGSL1	Down	7.26891
LMOD2	Up	4.89948	GPX3	Down	7.02045
DUSP6	Up	3.32944	GPR3	Down	6.23877
CLDN1	Up	3.16901	FLG	Down	5.84361
P15	Up	2.85767	A2M	Down	6.23877
SPRY4	Up	2.53669	PSG1	Down	3.07936
VDR	Up	2.50545	OLR1	Down	2.99577
ACSL5	Up	2.47184	RNF175	Down	2.95313
TNFRSF10B	Up	2.10256	GPM7	Down	2.56912
CTSL	Up	2.05574	BLCAP	Down	2.46682
TTBK2	Up	2.02018	ACSL3	Down	2.25696
PCSK1	Up	2.01142	WDR36	Down	2.12639

We next investigated whether LINC00152 silenced IL24 transcription by recruiting EZH2 or LSD1 to the IL24 promoter region. We used chromatin immunoprecipitation (ChIP) to determine the association of IL24 and EZH2 or LSD1. EZH2 was shown to bind the promoter region of IL24, while LINC00152 knockdown reduced the histone H3 lysine 27 trimethylation (H3K27me3) occupancy of the IL24 promoter locus (Fig. 5h). However, LSD1 did not bind the IL24 promoter locus (Additional file 5: Figure S3d). These results indicated that LINC00152 repression of target IL24 expression occured, at least partially, through interaction with EZH2.

### Ecotopic expression of IL24 inhibited LAD cell proliferation

**Fig. 6:**
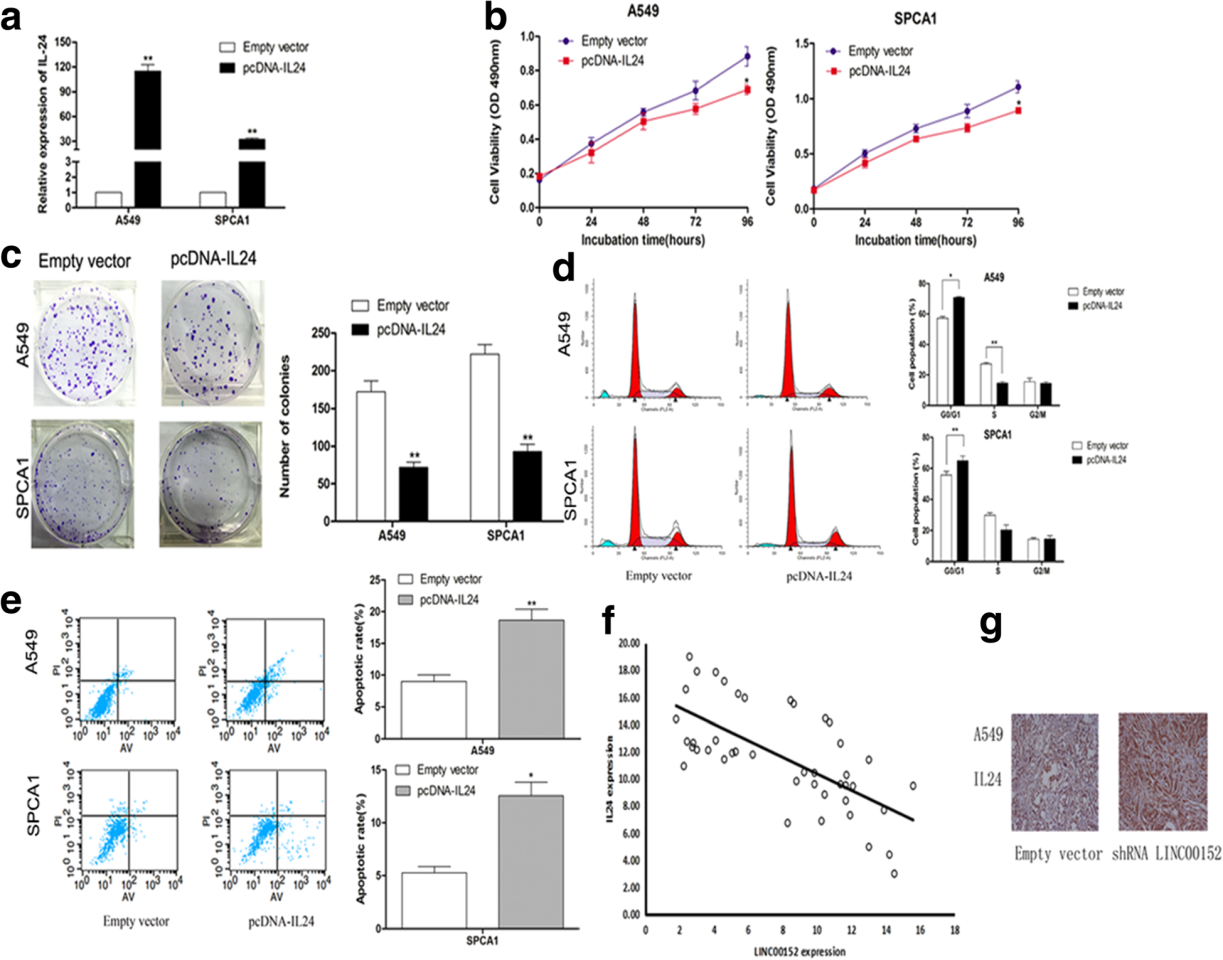
Effects of IL24 overexpression in A549 and SPCA1 cell in vitro. **a** IL24 expression in A549 and SPCA1 cells transfected with pcDNA3.1-IL24 vector. **b, c** MTT assays and colony formation assays were used to determine the cell viability. **d** Cell cycle was analyzed in A549 and SPCA1 cells. **e** Flow cytometry was performed to determine the apoptptic rates of cells. **f** Analysis of LINC00152 and IL24 mRNA levels in 43 paired LAD tissues. **g** The tumor sections were under IHC staining using antibodies against IL24. Values are shown as the mean ± s.d in three independent experiments. *P < 0.05, **P < 0.01

To investigate the functional role of IL24 in the LAD cell phenotype, we conducted gain-of-function assays. IL24 expression was greatly increased in A549 and SPCA1 cells following the transfection of a pcDNA3.1-IL24 vector (Fig. 6a + Additional file 1: Figure S1f). MTT and colony formation assays revealed that overexpression of IL24 impaired the proliferation ability of LAD cells (Fig. 6b, c). Additionally, flow cytometry analysis confirmed that IL24 upregulation increased G1/G0 arrest and induced cell apoptosis (Fig. 6d, e). Furthermore, IL24 expression was significantly negative correlated with LINC00152 expression (Fig. 6f), and the immunohistochemical analysis showed higher IL24 staining in tumors formed from A549 cells with stable LINC00152 knockdown (Fig. 6g). These results suggested that the ecotopic expression of IL24 inhibited cell proliferation and induces apoptosis in LAD cells.

**Fig. 7:**
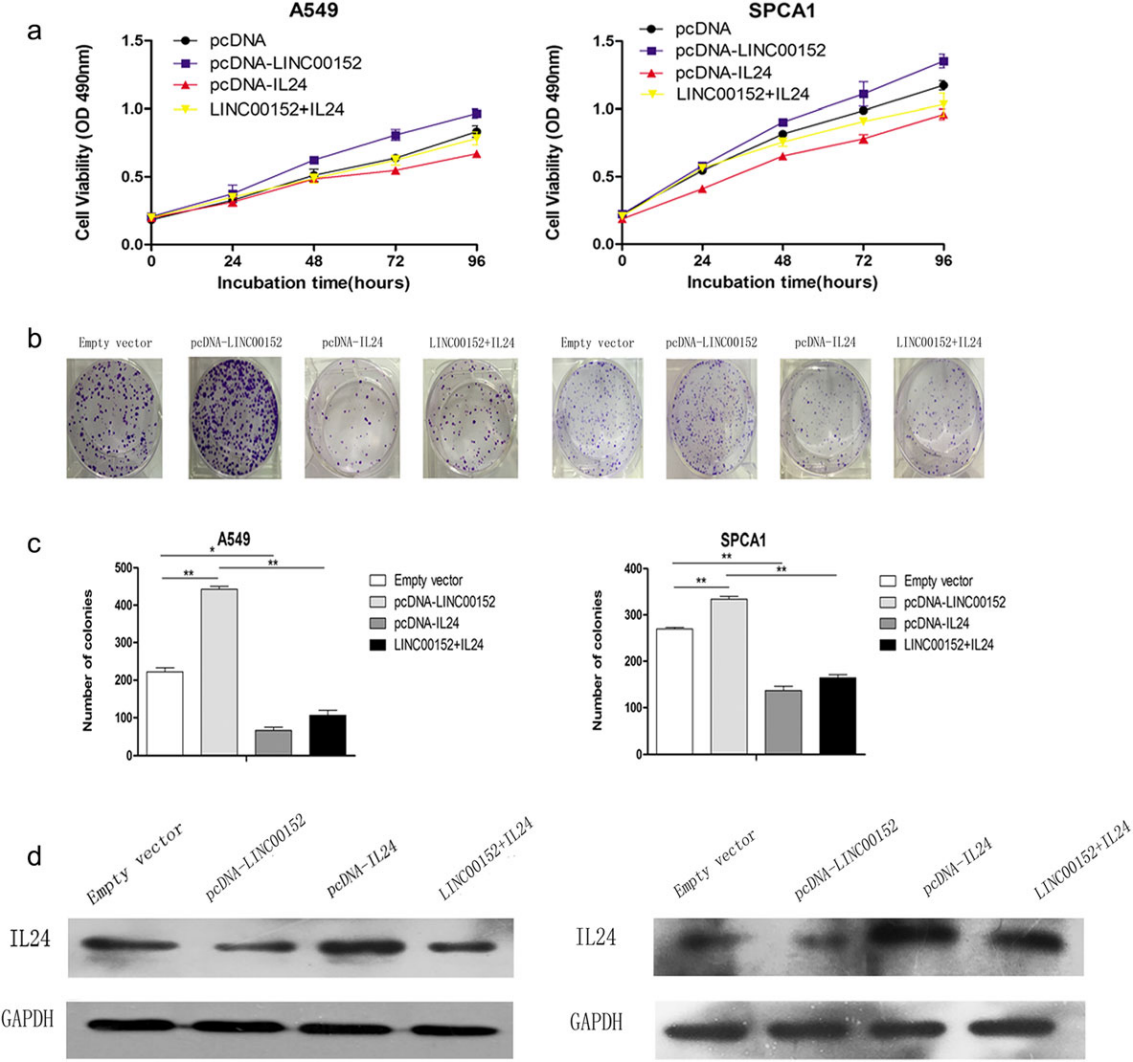
IL24 overexpression could partly reverse the oncogenic function of LINC00152. A549 and SPCA1 cells were transfected pcDNA3.1, pcDNA3.1-LINC00152, pcDNA3.1-IL24, pcDNA3.1-LINC00152 + IL24. **a–c** MTT assays and colony formation assays were used to determine the cell viability of transfected LAD cells. The colonies were counted and captured. **d** The IL24 expression was analyzed in western blotting. Values are shown as the mean ± s.d in three independent experiments. *P < 0.05, **P < 0.01

We also performed rescue assays to validate whether IL24 was involved in the LINC00152-mediated promotion of LAD cell proliferation. A549 and SPCA1 cells were co-transfected with pcDNA3.1-LINC00152 and pcDNA3.1-IL24. MTT analysis revealed that IL24 overexpression partially reversed the effects of LINC00152 overexpression-mediated promotion of LAD proliferation (Fig. 7a–c). Western blot analysis showed that IL24 expression levels was decreased in LINC00152 + IL24 cells compared with pcDNA3.1-IL24 transfected cells (Fig. 7d). Collectively, these data indicated that LINC00152 promoted LAD cell proliferation in part through the downregulation of IL24.

## Discussion

Recently, a number of studies have revealed crucial roles for lncRNAs in the development and progression of human cancers. In the case of LAD, several lncRNAs, such as SPRY4-IT1, LINC01133, AGAP2-AS1, and LINC00473, have been characterised and their function have been reported in NSCLC as well as underlying mechanisms [20–23]. We previously showed that lncRNA PVT1 is significantly upregulated in LAD tissues and cells, and that it promotes cell proliferation through epigenetically repressing tumor suppressor LATS2 expression by interacting with EZH2 [13]. In the present study, we identified another lncRNA, LINC00152, which is overexpressed in LAD tissues. Increased LINC00152 expression is associated with poor prognosis and shorter survival time of LAD patients. Moreover, knockdown of LINC00152 expression inhibited LAD cell proliferation, and induced cell apoptosis both in vitro and in vivo. These findings suggest that LINC00152 may exert an oncogenic function and play a key role in LAD development.

Previous studies demonstrated that LINC00152 is overexpressed in multiple cancers, including gastric cancer, HCC, and clear cell renal cell carcinoma. Ji et al. reported that LINC00152 is upregulated in HCC, and promotes cell proliferation in vitro and tumor growth in vivo through activating the mTOR pathway by binding to the promoter of the epithelial cell adhesion molecule gene [24]. Overexpression of LINC00152 also led to malignant biological behavior in clear cell renal cell carcinoma through promoting cell proliferation and invasion, and dramatically decreasing apoptosis [17]. Moreover, increased LINC00152 promoted cell growth and cell cycle progression in gastric cancer by repressing p15 and p21 transcription through binding EZH2 [16]. Consistent with these findings, the present study found that LINC00152 functions as an oncogene by promoting cell proliferation and anti-apoptosis in LAD cells, suggesting that it may be a common oncogenic lncRNA in human cancers.

Increasing evidence has revealed that lncRNAs contribute to tumorigenesis by silencing tumor suppressors or activating oncogenes through chromatin modification, genomic imprinting, RNA decay, and sponging miRNAs. We used RIP and RNA pulldown assays to show that LINC00152 directly binds EZH2 in LAD cells, suggesting that LINC00152 regulates underlying targets at the transcriptional level. Further RNA sequencing of LINC00152-knockdown LAD cells revealed that the tumor suppressor IL24 is a novel LINC00152 target in LAD cells. Interestingly, knockdown of EZH2 in LAD cells also upregulated IL24 expression, while ChIP assays showed that LINC00152 could recruit EZH2 to the IL24 promoter region and repress its transcription by mediating H3K27me3. These findings indicate that lncRNA LINC00152 plays a key role in EZH2-mediated repression of IL24 in LAD cells.

IL24, also known as melanoma differentiation-associated gene-7 (MDA-7), is a member of the IL10 cytokine family, and has been identified as an important immune mediator as well as a tumor suppressor [25]. Exogenous expression of IL24 by liposomes or adenoviruses specifically inhibited cancer cell growth and induced tumor-specific apoptosis in a broad spectrum of solid tumors, including breast cancer, neuroblastoma, and HCC [26–28]. IL24 was also shown to function as a tumor suppressor in hematological malignancies [25, 29] and to cause apoptosis by a secretory pathway in NSCLC [30], while IL24 overexpression suppressed migration and invasion in LAD [31]. In the present study, we also found that overexpression of IL24 in LAD cells significantly inhibited cell growth and induced cell apoptosis. Importantly, increased IL24 expression partially reversed the LINC00152 overexpression-induced promotion of growth in LAD cells, suggesting that the oncogenic role of LINC00152 may depend on IL24 regulation in LAD cells.

## Conclusion

We show for the first time that LINC00152 expression is upregulated in LAD tissues and cells, and that its overexpression is associated with poor prognosis and may be a prognostic factor for LAD patients. Knockdown of LINC00152 exerted tumor suppressive functions through inhibiting cell growth, and inducing cell apoptosis. Furthermore, LINC00152-mediated oncogenic effects occur in part through the epigenetic silencing of IL24 expression following binding with EZH2. We did not determine whether LINC00152 regulates other genes and mechanisms that underlie regulatory behavior, so further studies should be carried out to investigate this. Nevertheless, our findings strengthen the understanding of LAD pathogenesis, and facilitate the development of lncRNA-directed diagnostics and therapeutics against this disease.

## Additional files

Additional file 1: Table S2. Primer sequences. (XLSX 13 kb)
Additional file 2: Figure S1. LINC00152 expression decreased after LINC00152 silencing. (a) LINC00152 expression in A549 and SPCA1 cells transfected with three discrete chemically synthesized siRNAs. (b) LINC00152 expression in H1299 cells transfected with pcDNA3.1-LINC00152 vector. (c) Western blotting analysis of bcl-xl protein level in A549 and SPCA1 cells, GAPDH protein was used as an internal control. (d) EZH2 expression in A549 and SPCA1 cells transfected with si-EZH2. (e) Western blotting were performed to detect the EZH2 and LSD1 protein level in A549 and SPCA1 cells. (f) Western blotting analysis of IL24 protein level in A549 and SPCA1 cells, GAPDH protein was used as an internal control. Values are shown as the mean ± s.d in three independent experiments. **P* < 0.05, ***P* < 0.01. (TIF 6891 kb)
Additional file 3: Figure S2. Effects on tumor proliferation after LINC00152 overexpression in vivo. (a,b) H1299 cells transfected with empty vector or pcDNA-LINC00152 were injected into the nude mice (*n* = 3). Tumors before and after carrying from the nude mice. (TIF 5996 kb)
Additional file 4: Table S1. Microarray analysis. (XLS 1810 kb)
Additional file 5: Figure S3. LINC00152 could not recruit LSD1 to IL24 promoter. (a) LSD1 expression in A549 and SPCA1 cells transfected with si-LSD1. (b,c) qRT-PCR and western blotting were performed to detect the IL24 mRNA and protein level in A549 and SPCA1 cells. (d) ChIP-qRT-PCR of LSD1 occupancy and H3K4me2 binding in the IL24 promoter in A549 and SPCA1 cells treated with si-LINC00152(48h) or scrambled siRNA, IgG was used as a negative control. Values are shown as the mean ± s.d in three independent experiments. **P* < 0.05, ***P* < 0.01. (TIF 3427 kb)

## Abbreviations

ChIP: Chromatin immunoprecipitation
EZH2: Enhancer of zeste homolog 2
H3K27me3: Histone H3 lysine 27 trimethylation
HCC: Hepatocellular carcinoma
IL24: Interleukin 24
LAD: Lung adenocarcinoma
LINC00152: Long intergenic non-coding RNA 00152
lncRNAs: Long noncoding RNAs
MDA-7: Melanoma differentiation-associated gene-7
miRNAs: MicroRNAs
NSCLC: Non-small cell lung cancer
qRT-PCR: Quantitative reverse transcriptase Polymerase Chain Reaction
RIP: RNA immunoprecipitation
