# Supplementary figures and images for: Long intergenic non-coding RNA 00152 promotes lung adenocarcinoma proliferation via interacting with EZH2 and repressing IL24 expression
Source: Mol Cancer. 2017 Jan 21;16:17. doi: 10.1186/s12943-017-0581-3 (PMC5251237; doi:10.1186/s12943-017-0581-3)

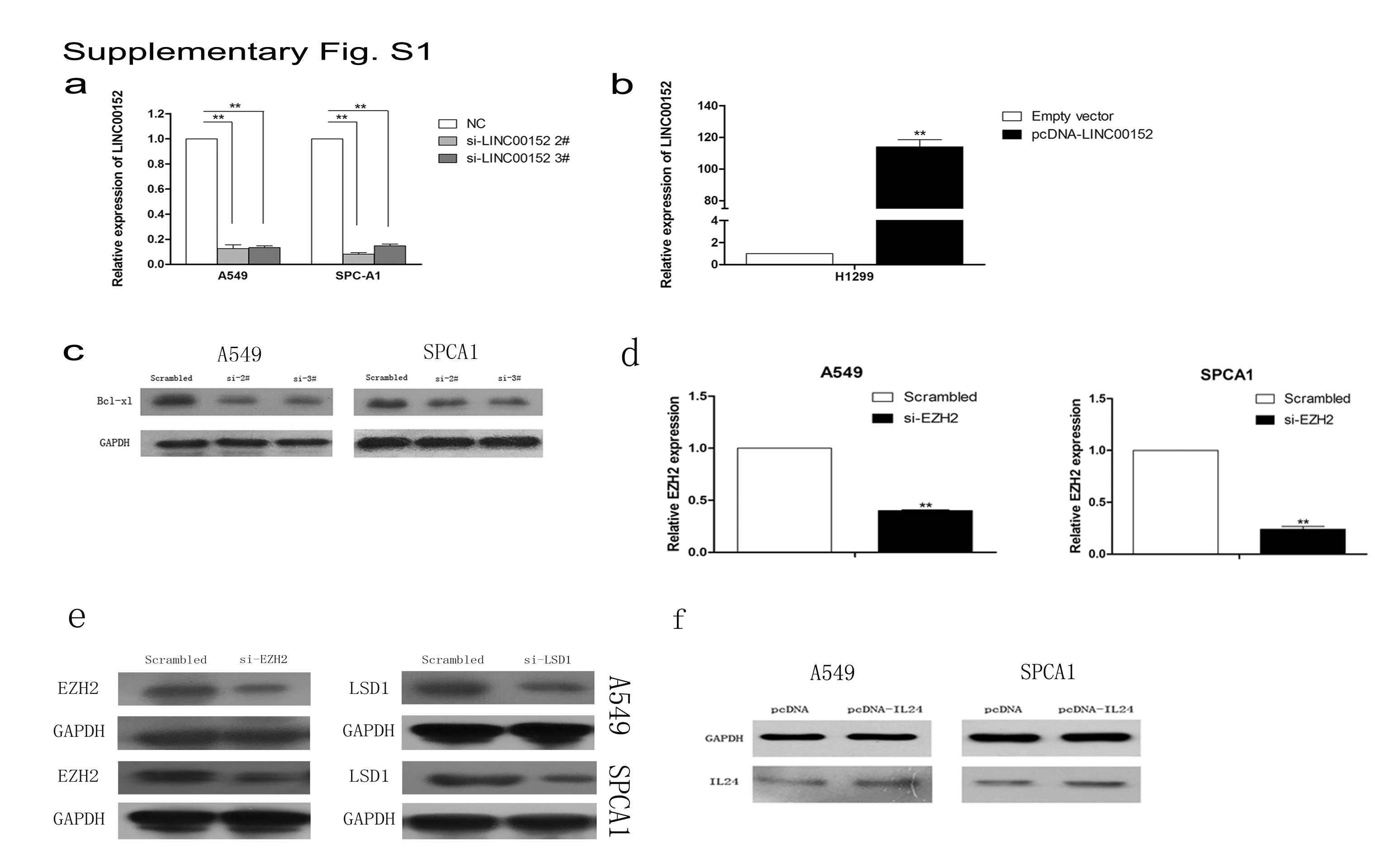

Supplement: Additional file 2: Figure S1. — LINC00152 expression decreased after LINC00152 silencing. (a) LINC00152 expression in A549 and SPCA1 cells transfected with three discrete chemically synthesized siRNAs. (b) LINC00152 expression in H1299 cells transfected with pcDNA3.1-LINC00152 vector. (c) Western blotting analysis of bcl-xl protein level in A549 and SPCA1 cells, GAPDH protein was used as an internal control. (d) EZH2 expression in A549 and SPCA1 cells transfected with si-EZH2. (e) Western blotting were performed to detect the EZH2 and LSD1 protein level in A549 and SPCA1 cells. (f) Western blotting analysis of IL24 protein level in A549 and SPCA1 cells, GAPDH protein was used as an internal control. Values are shown as the mean ± s.d in three independent experiments. *P < 0.05, **P < 0.01. (TIF 6891 kb) [file 12943_2017_581_MOESM2_ESM.tif]

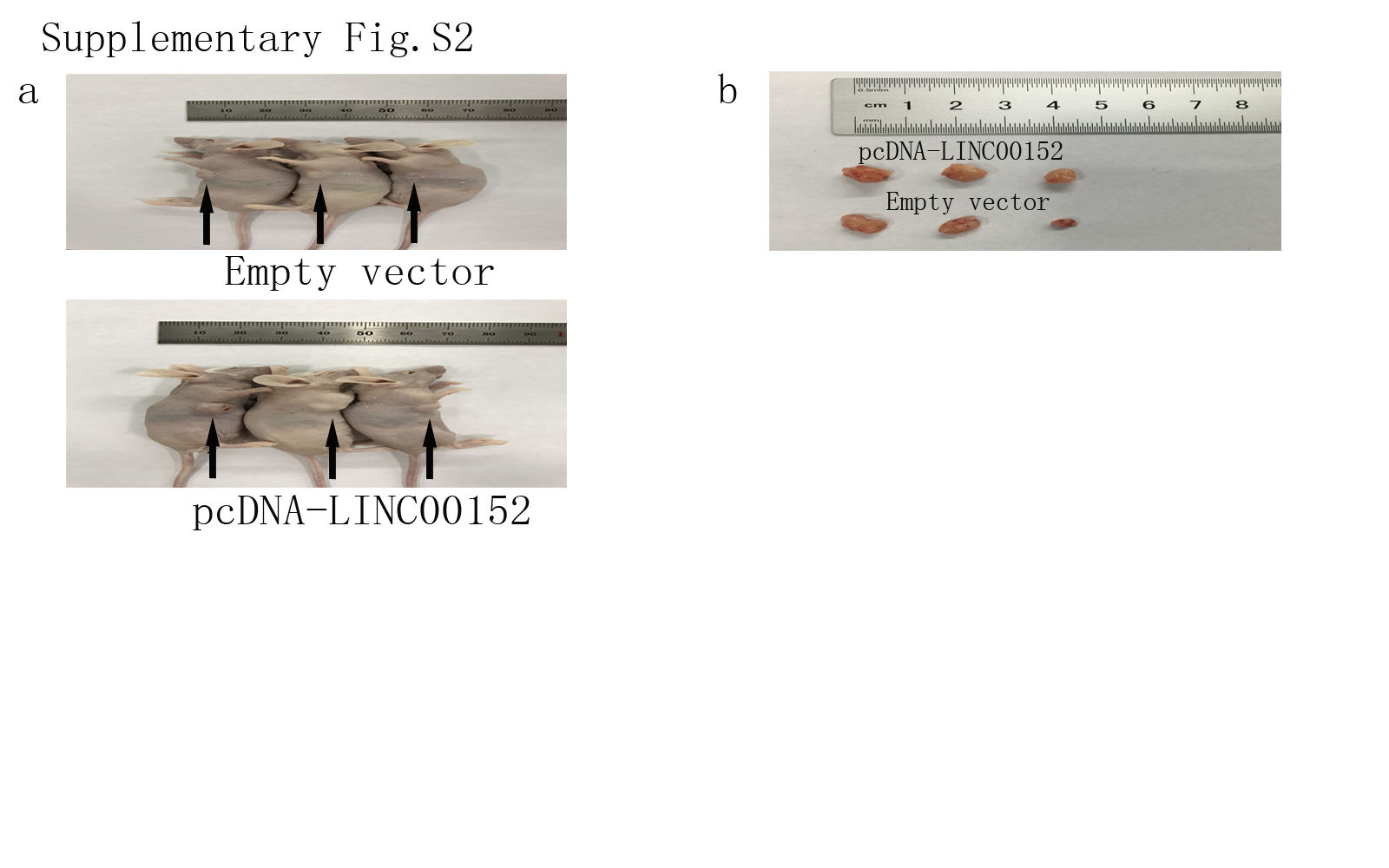

Supplement: Additional file 3: Figure S2. — Effects on tumor proliferation after LINC00152 overexpression in vivo. (a,b) H1299 cells transfected with empty vector or pcDNA-LINC00152 were injected into the nude mice (n = 3). Tumors before and after carrying from the nude mice. (TIF 5996 kb) [file 12943_2017_581_MOESM3_ESM.tif]

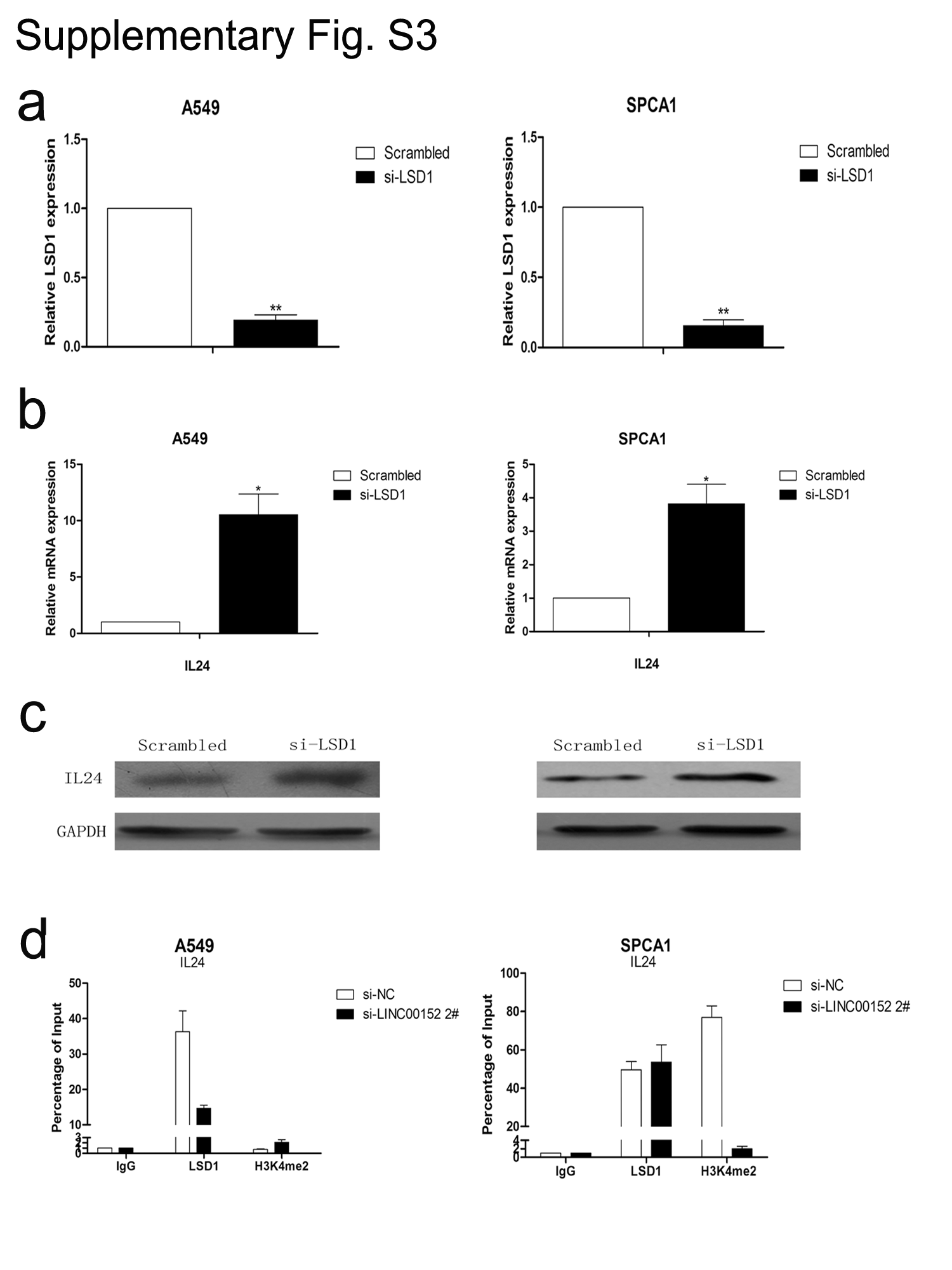

Supplement: Additional file 5: Figure S3. — LINC00152 could not recruit LSD1 to IL24 promoter. (a) LSD1 expression in A549 and SPCA1 cells transfected with si-LSD1. (b,c) qRT-PCR and western blotting were performed to detect the IL24 mRNA and protein level in A549 and SPCA1 cells. (d) ChIP-qRT-PCR of LSD1 occupancy and H3K4me2 binding in the IL24 promoter in A549 and SPCA1 cells treated with si-LINC00152(48h) or scrambled siRNA, IgG was used as a negative control. Values are shown as the mean ± s.d in three independent experiments. *P < 0.05, **P < 0.01. (TIF 3427 kb) [file 12943_2017_581_MOESM5_ESM.tif]
